# N-acetylcysteine Treatment Reduces Age-related Hearing Loss and Memory Impairment in the Senescence-Accelerated Prone 8 (SAMP8) Mouse Model

**DOI:** 10.14336/AD.2017.0930

**Published:** 2018-08-01

**Authors:** Aurore Marie, Johann Meunier, Emilie Brun, Susanna Malmstrom, Veronique Baudoux, Elodie Flaszka, Gaëlle Naert, François Roman, Sylvie Cosnier-Pucheu, Sergio Gonzalez-Gonzalez

**Affiliations:** ^1^CILcare, Parc Scientifique Agropolis, Montpellier, France; ^2^Amylgen, Montferrier-sur-Lez, France; ^3^Correlative Microscopy and Electron Tomography Platform, Hopital Saint Eloi, Montpellier, France

**Keywords:** SAMP8, hearing loss, antioxidant, N-acetylcysteine, aging

## Abstract

Age-related hearing loss (ARHL) is the most common sensory disorder in the elderly population. SAMP8 mouse model presents accelerated senescence and has been identified as a model of gerontological research. SAMP8 displays a progressive age-related decline in brain function associated with a progressive hearing loss mimicking human aging memory deficits and ARHL. The molecular mechanisms associated with SAMP8 senescence process involve oxidative stress leading to chronic inflammation and apoptosis. Here, we studied the effect of N-acetylcysteine (NAC), an antioxidant, on SAMP8 hearing loss and memory to determine the potential interest of this model in the study of new antioxidant therapies. We observed a strong decrease of auditory brainstem response thresholds from 45 to 75 days of age and an increase of distortion product amplitudes from 60 to 75 days in NAC treated group compared to vehicle. Moreover, NAC treated group presented also an increase of memory performance at 60 and 105 days of age. These results confirm that NAC delays the senescence process by slowing the age-related hearing loss, protecting the cochlear hair cells and improving memory, suggesting that antioxidants could be a pharmacological target for age-related hearing and memory loss.

Hearing loss is the most common form of sensory impairment in humans, affecting 360 million persons worldwide, with a prevalence of 183 million adult males and 145 million adult females. In nonsyndromic deafness, only hearing function is noticeably altered, whereas syndromic deafness is accompanied by other physiological defects. Although approximately 1 in 500 children are born with impaired hearing, sudden or progressive forms of hearing loss can manifest at any age [1].

The most common form of sensory impairment in elderly people is the age-related hearing loss (ARHL) [2]. This disorder is characterized by symmetric sensorineural hearing loss that starts at high frequencies with a prevalence of 35% of individuals over 65 years of age [3]. Although hearing loss has been considered to be part of a natural aging process, not all humans suffer from ARHL; genetic studies suggest that the source of variability is both genetic and environmental. Interestingly, statistical studies have established associations between age-related hearing loss and genes linked to reactive oxygen species (ROS) detoxification, such as arylamine *N*-acetyl-transferase 2 and glutathione *S*-transferase [4, 5], strongly confirming an essential role of mitochondria and oxidative stress in age-related hearing loss.

The senescence-accelerated prone 8 (SAMP8) mouse model is a strain derived from AKR/J mouse, selected for a phenotype toward accelerated senescence with a mean life span of 12 months [6]. SAMP8 mice have been identified as suitable for use as models in gerontological research [7]. This strain has been widely used in aging research to study phenotypes such as immune dysfunction, osteoporosis, and brain atrophy [6,7]. It has been reported that SAMP8 strain displays sequential degeneration of outer hair cells (OHCs), spiral ganglion neurons (SGNs), stria vascularis and ultimately inner hair cells (IHCs), which mimic human ARHL [8, 9]. Moreover, a recent study demonstrated that the molecular mechanisms associated with premature SAMP8 senescence involves oxidative stress, altered levels of anti-oxidant enzymes and decreased activity of Complexes I, II and IV which in turn lead to chronic inflammation and triggering of apoptotic cell death pathways [9].

High oxidative stress has been universally observed in SAMP8 and the age-related cochlear and central nervous system damages have also been directly associated to increase of ROS, mimicking human senescence. For this reason, we hypothesized that N-acetyl-cysteine, a potent antioxidant able to scavenge a big number of oxygen species after transformation to glutathione [10], could be an effective therapeutic agent to block the activation of age-related cell death mechanisms. To validate our hypothesis, we administrated NAC in the drinking water ad libitum and we analyzed auditory functions by auditory brainstem response (ABR) and distortion product otoacoustic emission (DPOAE), cochlea histological recovery by scanning electron microscopy (SEM) and memory impairment using object recognition test during ten weeks of treatment. We showed that daily NAC treatment induced otic- and neuro-protective effects in SAMP8 mouse model.

## MATERIAL AND METHODS

### Animals and drug administration

SAMP8/TaHsd female mice (weight 17-22 g), were obtained from Envigo at 3 weeks of age and maintained under specific pathogen-free conditions. Before starting the study, we allowed the mice to acclimatize for 7 days. Mice were kept in the animal facility in clear macrolon cages at 22±2°C, subjected to standard light cycles and with standards diet and water ad libitum. N-acetyl-L-cystine (Sigma Aldrich, A7250-50G) was diluted in the drinking water at 10 g/L and new solution was prepared every week during 10 weeks of treatment. The animals were treated with NAC from 30 to 105 days of age. All animal experiments were conducted in accordance with the European Directives (#2010/63/UE).

### Auditory brainstem response (ABR)

For ABR studies, mice were anesthetized using a mixture of ketamine (100 mg/kg) and xylazine (10 mg/kg) and the tragus was removed using sterile scissors in order to open the external auditory canal. Then, animals were placed in an acoustic chamber to completely isolate them from exterior noise and on a thermostatically regulated heating pad to maintain the temperature at 37°C. Animals were placed by group of three and earphones (Philips SHE8500) were disposed in the left ear of each mouse. An active electrode was placed in the vertex of the skull, a reference electrode under the skin of the mastoid bone and a ground electrode was placed in the neck skin. Sound waveforms were generated from Matlab, they consisted in tone pips of 2 milliseconds linear rise/fall times and no plateau, the stimuli were delivered at a rhythm of 20 per second. These were fed to TDT SA1 audio amplifiers which delivered their outputs to the earphones. The stimuli consisted of tone pip with center frequency of 8, 16, 20, 24 and 32 kHz, to cover the mice auditory frequency range. These series of tone pip were presented at various sound levels ranging from 90 to 10 dB with 10 dB steps. Evoked potentials were extracted by the signal averaging technique for each stimulus level. All biological signals were amplified through A-M system 1700 amplifiers with a gain of 10000 and filtered between 100 and 5 kHz. These signals were sent to analog/digital converter (CED Power 1401) and averaged several hundred times.

### Distortion product otoacoustic emission (DPOAE)

For DPOAE measures, mice were anesthetized using ketamine/xylazine mixture and a probe (OtoPhyLab) was inserted into the external left ear canal. The primary tone *F*_2_ was set at 1, 1.5, 2, 3, 4, 5, 8, 12, 16, 24 and 32 kHz. The frequency ratio *F*_2_/*F*_1_ was set at 1.2. Both *F*_2_ and *F*_1_ were given an intensity of 63 dB SPL. At all frequencies (*F*_2_), the input of DPOAE system received, digitized and evaluated the output of the microphone using the system software.

### Object recognition test

At the session 1, mice were placed individually in a squared open-field made in white plexiglass with a floor equipped with infrared light emitting diodes. Mice were habituated to the open-field during a 10-min duration session and their locomotor activity captured through an IR-sensitive camera and analyzed using the Ethovision software (Noldus).

24 hours after session 1, two identical objects were placed at defined positions. Each mouse was placed in the open-field and the exploratory activity was recorded during 10-min. The activity was analyzed in terms of number of contact with objects and duration of contacts. Finally, 24 h after session 2, the object in position #2 was replaced by a novel one differing in color, shape and texture from the familiar object. We choose this long-time interval of 24 h to assess the long-term memory which is altered in this mouse model. Each mouse was placed again in the open-field and the exploratory activity was recorded during a 10-min duration session. The preferential exploration index was calculated as the ratio of the number of contacts with the object in position #2 over the total number of contacts with the two objects [11, 12].

### Scanning electron microscopy

NAC and vehicle group mice were decapitated under deep anesthesia (ketamine/xylazine mixture) at 75 days of age and their cochleae were removed from the temporal bone and fixed in 2.5% glutaraldehyde in PHEM buffer (PIPES 60mM, HEPES free acid 25mM, EGTA 20 mM, MgCl2 2mM) overnight at room temperature. Then, the stria vascularis, tectorial, and Reissner’s membranes were removed. After rinsing in PHEM buffer, the samples were dehydrated in a graded series of ethanol (30-100%), critical point dried in CO_2_, coated with gold palladium, and observed using a Hitachi S4000 microscope [13].

**Figure 1.**
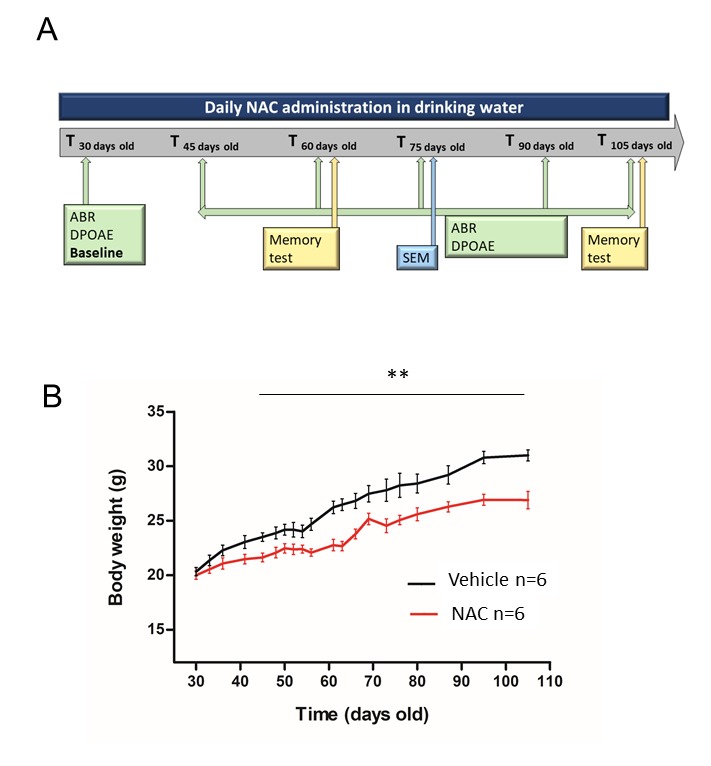
Experimental procedure and animal weight **A**) Schema of the experimental study. **B**) Mouse body weight evolution of NAC or vehicle treated group during the study. Data are shown as mean ± SEM. Significance was set at **p < 0.01. *n* = 6 mice by group.

**Figure 2.**
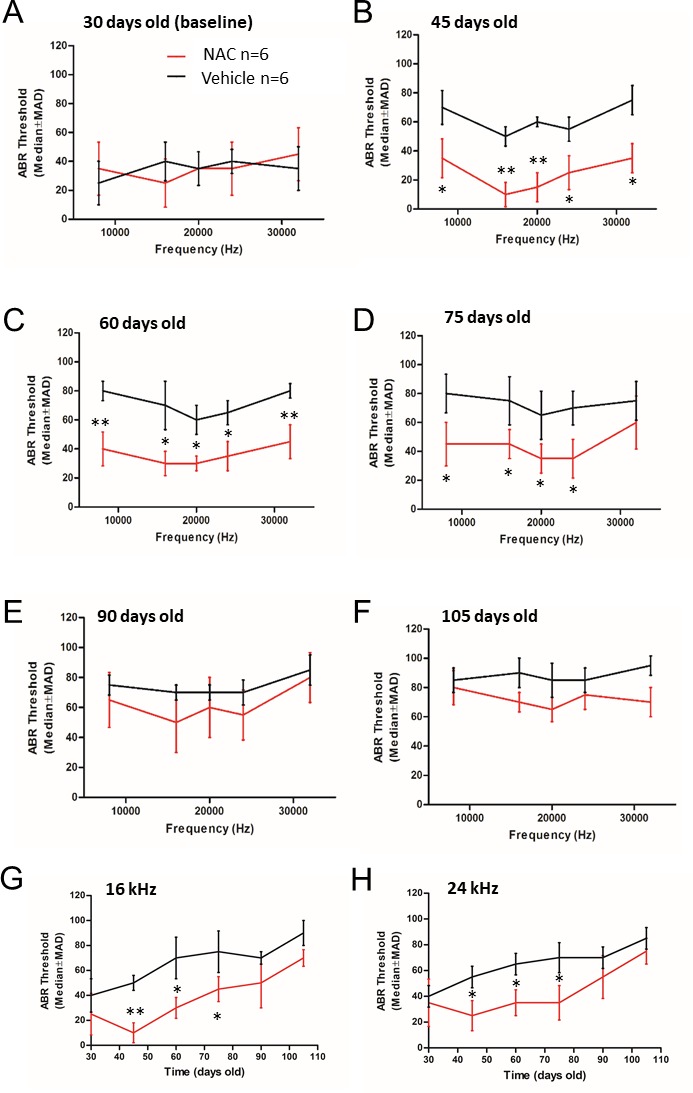
ABR thresholds representation Auditory Brain Response thresholds of NAC (red line) and vehicle (black) treated mice at 8, 16, 20, 24 and 32KHz at the indicated age (**A-F**). The age of mice is indicated on each the graph (days old). ABR thresholds of NAC (red line) and vehicle (black) treated mice at 16 kHz (**G**) and 24 KHz (**H**) during the time experiment. Data are shown as median ± MAD. Significance was set at *p < 0.05 and ** p < 0.01. *n* = 6 mice by group.

### Statistical analysis

Descriptive statistics by groups are provided with MEDIAN ± MAD (median ± Median Absolute Deviation) for ABR measures and with MEAN ± SEM (arithmetic mean ± Standard Error of the Mean) for animal body weight, DPOAE measures and memory test. A 2-way analysis of the covariance on ranks with repeated measurements on frequencies with animal’s baseline value as covariate was performed. A spherical correlation structure between frequency was used. Post-hoc analysis to compare NAC versus vehicle (water) group at each frequency was performed with the Dunnett p-value adjustment for multiplicity correction for ABR and DPOAE measures. 1-way ANOVA followed by t-test was performed for memory test analysis to compare NAC versus vehicle (water) group.

**Figure 3.**
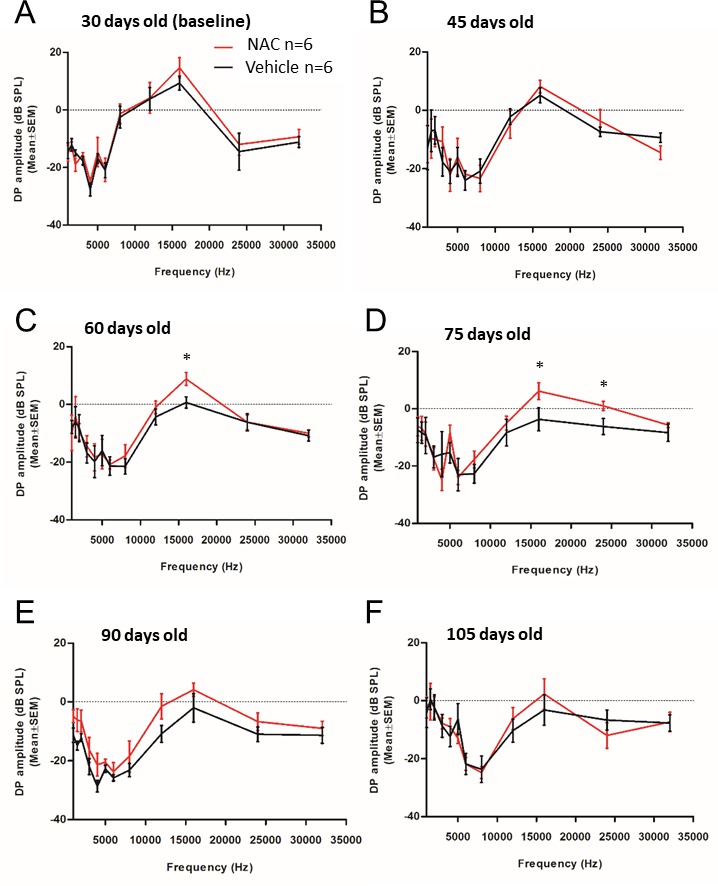
Distortion product otoacustic emission amplitude representation DPOAE amplitude level of NAC (red line) and vehicle (black line) at 1, 4, 6, 8, 10, 12, 16, 20, 25 and 32 kHz and an intensity of 63 dB SPL. The age of mice is indicated on each the graph (days old). Data are shown as mean ± SEM. Significance was set at *p < 0.05. *n* = 6 mice by group.

## RESULTS

### Decrease of body weight of NAC-treated SAMP8 group

To determine the influence of an antioxidant agent in age-related changes, we added NAC in the drinking water at 10g/L and we measured auditory functions for 10 weeks and memory impairment at 60 and 105 days old. NAC was administrated at 30 days of age, just after the first ABR threshold and DPOAE measures (baseline) and we stopped the treatment after the last ABR, DPOAE and memory test measures (at 105 days of age) (Fig. 1A). We also measured mouse body weight in order to determine the effect of daily NAC administration on body mass. We observed that NAC treated group presented a significant reduction of body weight compared to vehicle (water) group. NAC treated group reached a plateau of 26.9 ± 0.8 g at 105 days old whereas vehicle group presented a body weight of 31 ± 0.5 g the same age (Fig. 1B). This reduction could be explained by a role of NAC on eating and satiety regulation by increasing the activity of the cystine-glutamate antiporter as previously explained by Hurley and collaborators [14, 15].

**Figure 4.**
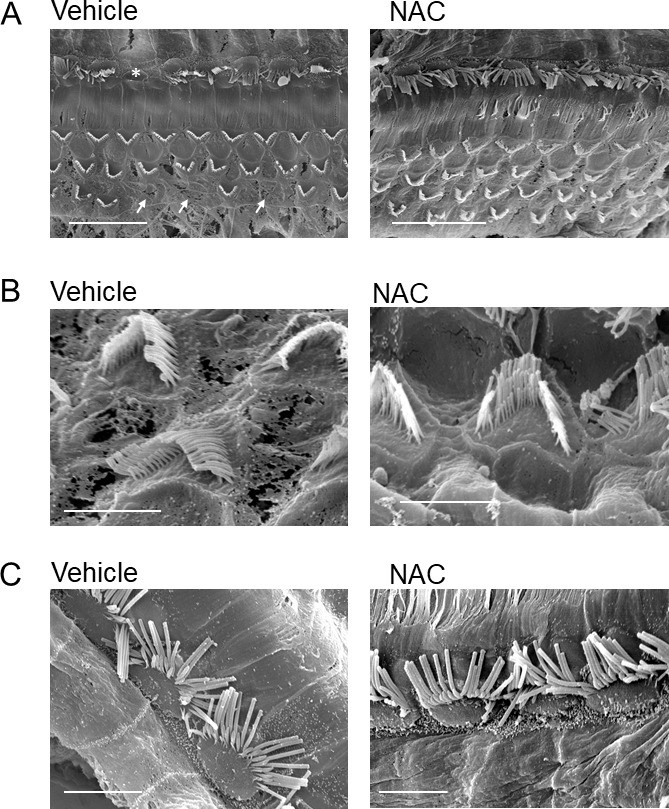
Scanning electron microscopy cochleae representative images of mice treated with NAC (right panels) and vehicle (left panels) **A**) Global view of middle part of cochlea showing the presence of IHC and OHC of treated mice. Arrows and asterisks mark the loss of OHC and IHC respectively. Scale bar = 15 µm. **B**) Magnification of OHC stereocilia of NAC and vehicle treated mice. Scale bar = 3 µm. **C**) Magnification of IHC stereocilia of NAC and vehicle treated mice. Scale bar = 5 µm. *n* = 3 mice by group. These images are representative images of 3 independent animals.

### NAC treatment reduces age-related ABR thresholds elevation in SAMP8 mice

ABRs are electric potentials recorded from scalp electrodes, and the first ABR wave represents the summed activity of the auditory nerve fibers contacting the IHCs. Using ABR technique, we previously demonstrated that SAMP8 strain presented a progressive ABR threshold increase from 45 days of age due to the accelerated degeneration of auditory system reaching a plateau at 148 days [16]. Here we observed that the ABR threshold values of NAC and vehicle groups were similar at baseline (30 days) (Fig. 2A). However, the ABR thresholds of NAC treated group were significantly lower from 45 to 75 days at all analyzed frequencies compared to vehicle-treated groups (Fig. 2B–D) and these ABR protective effects of NAC disappeared at 90 days of age (Fig. 2E–H). These results suggest that antioxidants are promising pharmacological candidates to delay the age-related hearing impairment.

### NAC treatment reduces age-related DPOAE amplitude decrease and protects hair cells in SAMP8 mice

On the other hand, DPOAEs are acoustic signals created and amplified by the cochlear epithelium offering an index of cochlear function and are linked to outer hair cell health (OHCs) [17] which amplify sound-evoked cochlear vibration. They do not depend on IHCs or auditory nerve fibers. It has long been established that DPOAE decrease in amplitude with increasing hearing loss [18] but there is also evidence that aging, independent of hearing loss, reduces otoacoustic emissions amplitude [19]. We observed that DPOAE amplitudes were similar at baseline (30 days of age) in NAC and vehicle treated groups (Fig. 3A) and the DPOAE amplitude of SAMP8 mice progressively decreased in vehicle group as we previously demonstrated [16]. However, the DPOAE amplitude of NAC treated mice were small but significantly higher at 16 kHz at 60 days of age (Fig. 3C) and at 16 and 24 kHz at 75 days of age (Fig. 3D) compared to vehicle-treated SAMP8 mice. Moreover, as observed in ABR threshold data, the effect of NAC on DPOAE was not observed anymore from 90 days (Fig. 3E, F) suggesting that NAC treatment was able to delay, but not to abolish, the loss of cochlear hair cell due to senescence process.

To corroborate these results, we analyzed cochlear hair cell presence using scanning electron microscopy at 75 days of age. We observed IHC and OHC loss in vehicle treated group whereas NAC treatment protected cochlear hair cells (Fig. 4A) corroborating the positive effect of NAC on DPOAE and ABR measures previously observed at the same age. However, no significant differences were observed in the morphology of IHC and OHC stereocilia between vehicle and NAC treated groups (Fig. 4B, C).

**Figure 5.**
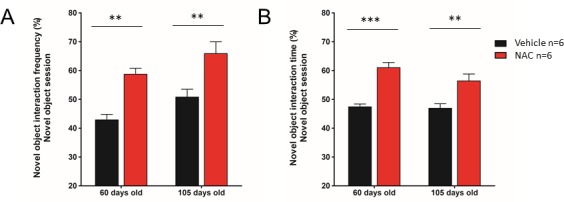
Object recognition test representation Working memory test of NAC and vehicle treated mice at 60 and 105 days old represented as % of frequency of interactions with the new object (**A**) and % of time spent with the new object (**B**) respectively. Data are shown as mean ± SEM. Significance was set at **p < 0.01 and ***p < 0.001. *n* = 6 mice by group.

### NAC treatment improves age-related memory loss without effects on anxiety

SAMP8 mice have been initially described as a model of age-related central nervous system degeneration and Alzheimer characterized by working and spatial memory impairment [20, 21], for this reason we determined the effect of NAC treatment in SAMP8 mouse memory using the object recognition test. It is well characterized that animals presenting memory deficits spend similar time and interaction number with both objects (around 50%) whereas healthy animals spend more time and interaction number with the new object (more than 60%) [11, 12]. In this way, we observed that NAC treated mice presented around 60% and 70% of interactions with the new object at 60 days and 105 days of age respectively, whereas vehicle groups remained around 40% and 50 % of interactions respectively (Fig. 5A). Moreover, the percentages of time spent with the new object were also higher in NAC treated mice compared to vehicle treated mice at these ages (Fig. 5B) confirming that NAC treatment significantly improved memory performance in SAMP8 mice at the early and advance stages of the senescence process.

## DISCUSSION

Here, using functional and histological studies, we demonstrated that daily NAC treatment was able to delay, but not to inhibit, the onset of hearing impairments due to accelerated senescence process. We also showed that NAC treatment improved memory in SAMP8 mice. To explain these results, we hypothesized that NAC reduced the overexpression of ROS produced by mitochondria leading to a reduction of cellular and mitochondrial damage and preventing cochlear hair cell and neuron apoptosis. Taken together, these data confirm that NAC induces otic- and neuro-protective effects in a model of accelerated senescence.

However, whereas the protective effect of NAC in ABR thresholds was strong, this effect remained moderate in DPOAE measures. It is well known that SAMP8 model presents a progressive degeneration of peripheral and central nervous system [6, 7] and because ABR represents the summed activity of neurons of nervous system we supposed that NAC mainly prevents neuronal degeneration explaining the strong effect in ABR thresholds protection. Moreover, corroborating this hypothesis we also observed that NAC significantly improved memory in SAMP8 suggesting that NAC was able to delay the degeneration of central nervous system neurons.

On the other hand, this effect of NAC is weaker in cochlear hair cells than neurons. We observed a protection of DPOAE amplitude decrease in two frequencies and this cochlear cell protection is also observed using SEM imaging. Nevertheless, we observed the same positive effects of NAC in ABR and DPOAE readouts from 45 to 75 days of age, showing a hearing improvement by neuronal and cochlear cell protection at the same time.

Finally, because at the end of the study (105 days old) ABR and DPOAE of vehicle and NAC treated mice are similar, we hypothesized that NAC treated mice presented a fast degeneration of IHCs and OHCs reaching a plateau of cell loss like vehicle treated mice and leading to the similar hearing loss in both groups.

Cochleae are extremely vulnerable to oxidative stress because of the high metabolic demands of their mechanosensory hair cells in response to sound stimulation. Normally, ROS produced by hair cell mitochondria during physiological conditions are scavenged by hair cell endogenous antioxidant mechanisms. However, in aging conditions, the expression of ROS scavenger enzymes decreases and the oxidative species unbalance leading to the increase of oxidation and genetic and cellular alterations which cause cellular dysfunctions and progressive cochlear degeneration [22]. This increase of ROS also induces impaired blood flow to the cochlea, inflammation and degeneration of supporting structures and nerve fibers [23].

In this way, antioxidants such as glutathione, d-methionine, resveratrol and ascorbic acid, all attenuated noise-induced hearing loss in animal models when given before noise exposure [24]. Interestingly, polymorphisms in the gene encoding catalase have been linked to an increased susceptibility to hearing loss in humans, and mice that are heterozygous for a mutation in *Sod1* gene show an increased vulnerability to hearing loss induced by noise exposure [25]. These genetic findings provide additional evidence that antioxidant treatment might be crucial for the maintenance and recovery of normal hearing under loud noise conditions. Taken together, these data suggest that the therapy focusing in the reduction of this increase of oxidative stress in cochlea, including the enzymes that are involved in glutathione metabolism and in the breakdown of superoxide anions and hydrogen peroxidase, could be feasible options for the treatment of several types of hearing loss.

Corroborating this hypothesis, some clinical and military trials have been carried out for temporary threshold shift, in which administration of antioxidant nutritional supplements before moderate noise exposure showed some beneficial effects [26, 27]. However, clinical trials using NAC remain presently controversial and inconclusive. Whereas Kramer and collaborators published that NAC treatment didn’t provide protection from temporary thresholds shifts after noise exposure [28], Kopke and collaborators demonstrated that NAC significantly reduced auditory threshold shifts and DPOAE changes in military subjects undergoing routine weapons training [29]. However, whereas our results suggest a preventive effect of NAC in the age-related senescence process until now no long-term preventive clinical studies have been carried out in humans.

For this reason, the development of new antioxidant compound is essential to understand the role of oxidative stress in hearing impairment and to prevent age-related hearing loss in the future.

## References

[1] Thompson DC, McPhillips H, Davis RL, Lieu TL, Homer CJ, Helfand M (2001). Universal newborn hearing screening: summary of evidence. JAMA, 286, 2000-2010. 11667937 10.1001/jama.286.16.2000

[2] Someya S, Prolla TA (2010). Mitochondrial oxidative damage and apoptosis in age-related hearing loss. Mech Ageing Dev, 131:480-486. 20434479 10.1016/j.mad.2010.04.006 PMC4086639

[3] Yamasoba T, Lin FR, Someya S, Kashio A, Sakamoto T, Kondo K (2013). Current concepts in age-related hearing loss: epidemiology and mechanistic pathways. Hear Res, 303, 30-38. 23422312 10.1016/j.heares.2013.01.021 PMC3723756

[4] Unal M, Tamer L (2005). N-acetyltransferase 2 gene polymorphism and presbycusis. Laryngoscope, 115:2238-41 16369173 10.1097/01.mlg.0000183694.10583.12

[5] Bared A, Ouyang X, Angeli S, Du LL, Hoang K, Yan D, Liu XZ (2010). Antioxidant enzymes, presbycusis, and ethnic variability. Otolaryngol. Head Neck Surg, 143, 263-268. 10.1016/j.otohns.2010.03.024 PMC2913419 20647132

[6] Takeda T, Matsushita T, Kurozumi M, Takemura K, Higuchi K, Hosokawa M (1997). Pathobiology of the Senescence-accelerated Mouse (SAM). Experimental Gerontology, 32:117-127. 9088909 10.1016/s0531-5565(96)00068-x

[7] Tomobe K, Nomura Y (2009). Neurochemistry, neuropathology, and heredity in SAMP8: a mouse model of senescence. Neurochem Res, 34(4):660-9. 19247832 10.1007/s11064-009-9923-x

[8] Hosokawa M, Ueno M (1999). Aging of blood-brain barrier and neuronal cells of eye and ear in SAMP mice. Neurobiol Aging, 20(2):117-23. 10537021 10.1016/s0197-4580(99)00029-9

[9] Menardo J, Tang Y, Ladrech S, Lenoir M, Casas F et al (2012). Oxidative stress, inflammation, and autophagic stress as the key mechanisms of premature age-related hearing loss in SAMP8 mouse Cochlea. Antioxid Redox Signal, 16(3):263-74. 21923553 10.1089/ars.2011.4037

[10] Sen CK (2001). Antioxidant and redox regulation of cellular signaling: introduction. Med Sci Sports Exerc, 33:368-370 11252060 10.1097/00005768-200103000-00005

[11] Maurice T, Roman FJ, Su TP, Privat A (1996). Beneficial effects of sigma agonists on the age-related learning impairment in the senescence-accelerated mouse (SAM). Brain Res, 733(2):219-30. 8891305 10.1016/0006-8993(96)00565-3

[12] Meunier J, Villard V, Givalois L, Maurice T (2013). The γ-secretase inhibitor 2-[(1R)-1-[(4-chlorophenyl) sulfonyl](2,5-difluorophenyl) amino] ethyl-5-fluorobenzenebutanoic acid (BMS-299897) alleviates Aβ1-42 seeding and short-term memorydeficits in the Aβ25-35 mouse model of Alzheimer’s disease. Eur J Pharmacol, 698(1-3):193-9. 23123349 10.1016/j.ejphar.2012.10.033

[13] Ladrech S, Wang J, Simonneau L, Puel JL, Lenoir M (2007). Macrophage Contribution to the Response of the Rat Organ of Corti to Amikacin. J Neuros Res, 85:1970-1979 10.1002/jnr.21335 17497672

[14] Hurley MM, Resch JM, Maunze B, et al (2016). N-acetylcysteine (NAC) decreases binge eating in a rodent model. Int J Obes (Lond), 40(7): 1183-1186 26975440 10.1038/ijo.2016.31 PMC4935583

[15] McClure EA, Gipson CD, Malcolm RJ, Kalivas PW, Gray KM (2014). Potential Role of N-Acetylcysteine in the Management of Substance Use Disorders. CNS drugs, 28(2):95-106. 24442756 10.1007/s40263-014-0142-x PMC4009342

[16] Marie A, Larroze-Chicot P, Cosnier-Pucheu S, Gonzalez-Gonzalez S (2017). Senescence-accelerated mouse prone 8 (SAMP8) as a model of age-related hearing loss. Neurosciences Letters, 656:138-143. 10.1016/j.neulet.2017.07.037 28739348

[17] Kemp DT (2002). Otoacoustic emissions, their origin in cochlear function, and use. Br Med Bull, 63223-241. 10.1093/bmb/63.1.223 12324396

[18] Lonsbury-Martin BL, Martin GK (1990). The clinical utility of distortion-product otoacoustic emissions. Ear Hear, 11144-154. 10.1097/00003446-199004000-00009 2187725

[19] Uchida Y, Ando F, Shimokata H, Sugiura S, Ueda H, Nakashima T (2008). The effects of aging on distortion-product otoacoustic emissions in adults with normal hearing. Ear Hear, 29(2):176-84. 18595184 10.1097/aud.0b013e3181634eb8

[20] Akiguchi I, Pallàs M, Budka H, Akiyama H, Ueno M, et al (2017). SAMP8 mice as a neuropathological model of accelerated brain aging and dementia: Toshio Takeda’s legacy and future directions. Neuropathology, doi: 10.1111/neup.12373. 28261874

[21] Wang J, Cheng X, Zeng J et al (2017). LW-AFC Effects on N-glycan Profile in Senescence-Accelerated Mouse Prone 8 Strain, a Mouse Model of Alzheimer’s Disease. Aging Dis, 1; 8(1):101-114. 28203484 10.14336/AD.2016.0522 PMC5287383

[22] Fujimoto C, Yamasoba T (2014). Oxidative Stresses and mitochondrial dysfunction in age-related hearing loss. Oxid Med Cell Longev, 582-849. 10.1155/2014/582849 PMC4106174 25110550

[23] Shi X, Nuttall AL (2003). Upregulated iNOS and oxidative damage to the cochlear stria vascularis due to noise stress Brain Res, 967(1-2):1-10. 10.1016/s0006-8993(02)04090-8 12650960

[24] Tavanai E, Mohammadkhani G (2017). Role of antioxidants in prevention of age-related hearing loss: a review of literature. Eur Arch Otorhinolaryngol, 274(4):1821-1834 27858145 10.1007/s00405-016-4378-6

[25] Johnson KR, Yu H et al (2010). Separate and combined effects of Sod1 and Cdh23 mutations on age-related hearing loss and cochlear pathology in C57BL/6J mice. Hear Res, 268(1-2):85-92. 20470874 10.1016/j.heares.2010.05.002 PMC2923272

[26] Attias J, Bresloff I, Haupt H, Scheibe F, Ising H (2003). Preventing noise induced otoacoustic emission loss by increasing magnesium (Mg2+) intake in guinea-pigs. J Basic Clin Physiol Pharmacol, 14(2):119-36. 14558727 10.1515/jbcpp.2003.14.2.119

[27] Quaranta A, Scaringi A, Bartoli R, Margarito MA, Quaranta N (2004). The effects of ’supra-physiological’ vitamin B12 administration on temporary threshold shift. Int J Audiol, 43(3):162-5. 15198380 10.1080/14992020400050022

[28] Kramer S, Dreisbach L, Lockwood J, Baldwin K, Kopke R, Scranton S, O’Leary M (2006). Efficacy of the antioxidant Nacetylcysteine (NAC) in protecting ears exposed to loud music. J Am Acad Audiol, 17(4), 265-278. 16761701 10.3766/jaaa.17.4.5

[29] Kopke R, Slade MD, Jackson R, Hammill T, Fausti S (2015). Efficacy and safety of N-acetylcysteine in prevention of noise induced hearing loss: a randomized clinical trial. Hear Res, 323:40-50. 25620313 10.1016/j.heares.2015.01.002

